# Adverse Drug Event Reporting From Clinical Care: Mixed-Methods Analysis for a Minimum Required Dataset

**DOI:** 10.2196/10248

**Published:** 2018-06-28

**Authors:** David Peddie, Serena S Small, Katherin Badke, Chantelle Bailey, Ellen Balka, Corinne M Hohl

**Affiliations:** ^1^ Centre for Clinical Epidemiology and Evaluation Vancouver Coastal Health Research Institute Vancouver, BC Canada; ^2^ School of Communication Simon Fraser University Burnaby, BC Canada; ^3^ Vancouver General Hospital Department of Pharmaceutical Sciences Vancouver, BC Canada; ^4^ Department of Emergency Medicine University of British Columbia Vancouver, BC Canada; ^5^ Vancouver General Hospital Emergency Department Vancouver, BC Canada

**Keywords:** adverse drug event, adverse drug reaction, data fields, dataset, reporting, pharmacovigilance, mixed-methods, clinician-informed design

## Abstract

**Background:**

Patients commonly transition between health care settings, requiring care providers to transfer medication utilization information. Yet, information sharing about adverse drug events (ADEs) remains nonstandardized.

**Objective:**

The objective of our study was to describe a minimum required dataset for clinicians to document and communicate ADEs to support clinical decision making and improve patient safety.

**Methods:**

We used mixed-methods analysis to design a minimum required dataset for ADE documentation and communication. First, we completed a systematic review of the existing ADE reporting systems. After synthesizing reporting concepts and data fields, we conducted fieldwork to inform the design of a preliminary reporting form. We presented this information to clinician end-user groups to establish a recommended dataset. Finally, we pilot-tested and refined the dataset in a paper-based format.

**Results:**

We evaluated a total of 1782 unique data fields identified in our systematic review that describe the reporter, patient, ADE, and suspect and concomitant drugs. Of these, clinicians requested that 26 data fields be integrated into the dataset. Avoiding the need to report information already available electronically, reliance on prospective rather than retrospective causality assessments, and omitting fields deemed irrelevant to clinical care were key considerations.

**Conclusions:**

By attending to the information needs of clinicians, we developed a standardized dataset for adverse drug event reporting. This dataset can be used to support communication between care providers and integrated into electronic systems to improve patient safety. If anonymized, these standardized data may be used for enhanced pharmacovigilance and research activities.

## Introduction

Patients commonly transition between health care locations and care providers. Yet, electronic medical records containing critical information about a patient’s medical care are usually confined to one health care sector within a geographic location (eg, a hospital) or to a group of care providers who share a common office or the same specialty (eg, a family physician group practice) [1]. National and international health care accreditation bodies and patient safety organizations have emphasized the importance of transferring accurate medication histories to avoid unintentional errors and patient harm when transitions between care locations or care providers occur [2,3]. These are reflected in the established standards and goals for obtaining and documenting the best possible medication histories [2,4-7].

Despite significant progress in this area, information sharing about adverse drug events (ADEs) remains inadequate, even though these are the leading cause of emergency department visits and hospitalizations [8-10]. Emerging evidence suggests that inadequate information sharing about ADEs across health care sectors and between care providers may lead to unintentional re-exposures of patients to medications that previously caused harm [11]. In a recent large cohort study of elderly patients in Ontario, Canada, 55% of patients hospitalized for a fall-related injury while on high-risk medications were re-exposed to the same medication—a benzodiazepine or a neuroleptic—within 180 days, most within only 90 days [12]. This study also found that 38% of the elderly who were admitted for hypoglycemia while on glyburide were restarted on the same medication, despite the medication-associated risk of hypoglycemia in this age group and the availability of safer treatment options [12]. These data indicate that a gap exists in information continuity about medication safety risks that has the potential to cause harm when patients transition between care locations and providers [13].

Electronic systems could enable more accurate and complete documentation of medication safety risks and play a pivotal role in electronically communicating this information to other care providers to close this gap, but they are presently underutilized for this purpose [14]. Many existing electronic medical record systems include data fields for allergy documentation. However, the structure of allergy data collection modules is inappropriate for the documentation of many common ADEs (eg, drug-disease state interactions). Even when broader fields are available to document ADEs, these are most commonly in free text format and, therefore, unstructured and nonstandardized, making them prone to misinterpretation when read by other care providers. Electronic ADE reporting systems in use by pharmacovigilance organizations (eg, Health Canada’s MedEffect program) contain more structured and standardized data entry fields, but are burdensome to clinicians as they are external to electronic medical record systems and request clinically irrelevant information (eg, lot numbers of vaccines). ADE reporting within these systems is designed solely for drug regulatory purposes and is disconnected from clinical care activities, such that many clinicians do not access the systems at all [15-17].

Our objective was to develop a set of standardized data fields that clinicians could use to document and share information about ADEs from the point-of-care to address the information needs of clinicians and the limitations of existing systems. A secondary objective was to understand how electronic ADE documentation could be integrated into clinical activities to minimize the burden of documentation while improving reporting consistency, accuracy, and quantity.

## Methods

### Design and Setting

This was a mixed-methods study completed in British Columbia (BC), Canada, between March 2014 and April 2016 using a phased approached. As we have previously published our research methodology [15] and the results of some individual phases of this work [16,18], in this manuscript, we have focused on the research results used to develop, refine, and prioritize a minimum required dataset for ADE reporting.

In the first phase, we completed a systematic review of the existing ADE reporting systems [16]. We used information derived from our systematic review to develop a preliminary dataset that we presented to clinicians in iterative workshops in order to understand which data fields should be integrated into the minimum required dataset, their priority for integration, as well as their reporting sequence [15]. In parallel, we completed qualitative fieldwork to inform our understanding of the clinical nature of ADEs, clinicians’ workflow in diagnosing ADEs, and challenges related to their documentation [19-21]. This informed our design decisions and will be integral to the successful implementation of the set of data fields [21]. We then pilot-tested a paper-based ADE data collection form in two clinical settings and refined the final dataset [18].

The University of British Columbia Research Ethics Board reviewed and approved the study protocol. Workshop participants provided implied consent, and care providers observed during workplace observations and pilot testing provided verbal consent. Consolidated criteria for reporting qualitative research informed the reporting of study findings.

### Systematic Review

We began our work by completing a qualitative systematic review to synthesize data fields from existing ADE reporting systems worldwide [15,16]. We worked with a professional librarian to complete a systematic electronic bibliographic reference database and electronic gray literature search to identify ADE reporting systems worldwide. We developed, piloted, and refined a standardized data collection form to extract data about the reporting concepts and data fields used in each reporting system.

After identifying ADE reporting concepts and data fields, we imported them into the visual thinking software Inspiration 9.2 (Multimedia Appendix 1). We represented each individual data field with a bubble. Three research assistants (CB, DP, and MW) removed duplicates for identical data fields (eg, labeled “suspect medication”) and summarized their frequency by indicating the number of instances that the data field was used by all reporting systems. We then sorted the remaining bubbles into categories representing broad reporting concepts. In the third step, we collapsed nearly identical data fields (eg, “suspect medication” and “suspected medication”) and identified the relationships and hierarchies between reporting concepts and data fields. This allowed us to create a preliminary reporting form containing all data elements and concepts used in ADE reporting internationally.

### Qualitative Fieldwork

In order to understand the limitations of and existing means of documenting ADEs in clinical practice, we completed qualitative observations of care providers. Trained research assistants (CB, DP, SSS, and MW) observed clinical pharmacists and physicians in emergency departments and wards in 4- to 8-hour shifts at various times of the day and days of the week. We recruited a convenience sample of participants via email, word of mouth, and personal connections of care providers on the research team. Research assistants observed the process of patient care, which included clinicians managing patients’ medications and occasionally investigating, documenting, and treating ADEs. We sought to understand the real-world clinical experiences related to ADEs, recognizing that retrospective accounts of ADEs may gloss over important characteristics, challenges, and work activities. We aimed to produce nuanced accounts of clinicians’ workflows—patterns in their activities and interactions with patients and other clinicians and artifacts in the care setting (eg, electronic medical records, paper charts, forms). We used the findings from our observations to inform design decisions, particularly in relation to the implementation of the set of data fields. Two research assistants (DP and SSS) coded the field notes from the observations using qualitative data analysis software (NVivo 11). Following initial inductive coding, the team met regularly to discuss emergent results and finalize a coding structure.

### Workshops

**Figure 1:**
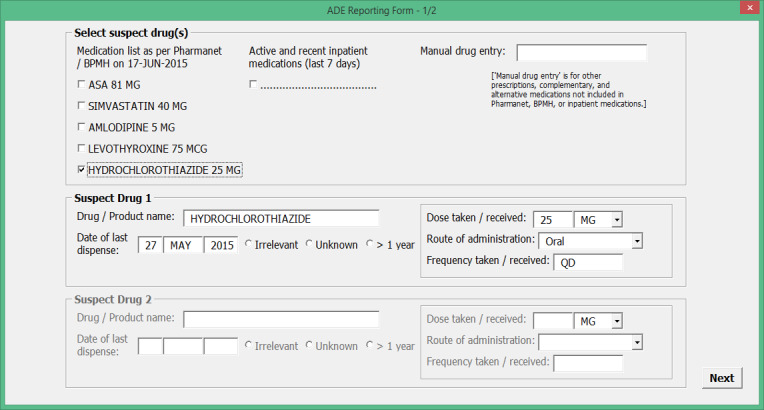
Sample screenshot from a preliminary adverse drug event (ADE) reporting form created using Microsoft Visual Basic for Applications.

**Figure 2:**
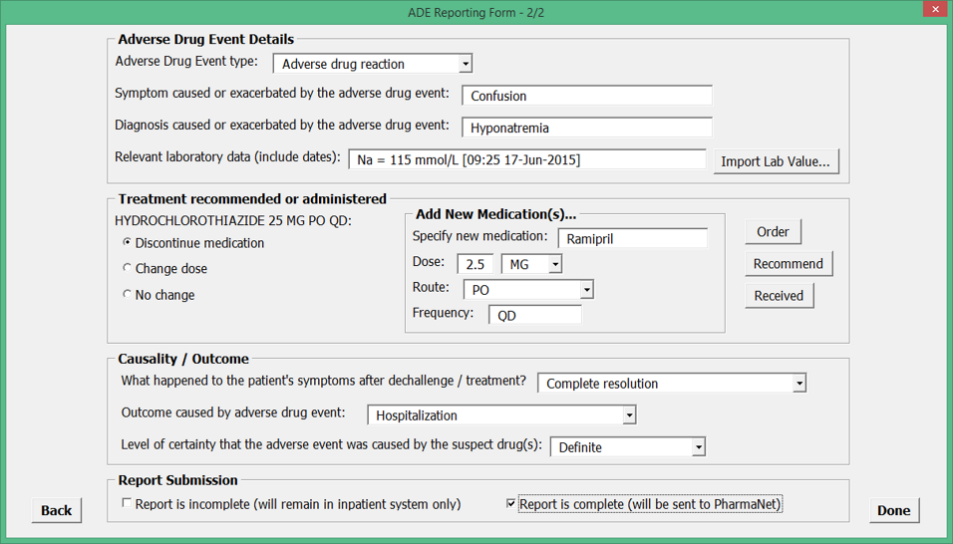
Sample screenshot from a preliminary adverse drug event (ADE) reporting form created using Microsoft Visual Basic for Applications.

To obtain feedback on our preliminary ADE data field set, we created a preliminary reporting form using Microsoft Visual Basic for Applications to resemble a screenshot from a computer (Figures 1 and 2). The preliminary form contained all of the reporting concepts identified in the systematic review. We developed data formats and value sets for different data fields by drawing on existing standards.

We presented this preliminary form to groups of clinicians in workshops scheduled during lunchtime rounds for clinicians practicing in hospital settings and scheduled in the evening for clinicians practicing outside of hospitals. We targeted groups of hospital- and community-based pharmacists, emergency department physicians, general practitioners, and hospitalists as these individuals commonly diagnose, treat, or follow up patients with acute ADEs. We recruited prospective participants using posters, email invitations, and in-person conversations with colleagues. The sessions were led or co-led by a practicing physician (CMH) or clinical pharmacist (KB) on the team and were attended by research assistants (DP and SSS) who took field notes during the sessions. We informed participants that our principal goal was to design a novel system to document and report ADEs and to obtain their feedback on our preliminary form. We presented ADE cases that we had observed in prior qualitative fieldwork to facilitate the discussion. We asked participants to identify information about the event that they felt was, or was not, required and how and where the information should be documented within the form. We asked participants to contemplate the required information needs from the perspectives of someone needing to document the information as well as receiving the report in order to balance the need to minimize documentation while ensuring that the required information was available.

Following each workshop, our team revised the data fields to incorporate feedback, producing a refined data field set for the next meeting or group. We maintained a log of changes that were made to the form, including the rationale for each change. Two research assistants (DP and SSS) coded the field notes from the workshops using the same approach as the qualitative observations. We considered the form to be a draft data field set when no novel suggestions or concerns were raised.

### Pilot Testing

A research assistant (AC) piloted the form in two clinical settings to test it for content and functionality prior to its planned computerization [18]. During paper-based pilot testing, we sought to understand the electronic linkages that would be required in other systems to facilitate reporting by observing which information sources clinicians accessed when they completed the form. The trained research assistant observed clinicians using “lightweight ethnography” supplemented with semistructured interviews. Lightweight ethnography is a method that enables the collection of specific relevant information while acknowledging that a complete comprehension of the work setting is not possible or necessary [22]. We recruited a convenience sample of clinical pharmacists through email invitations and in-person conversations with colleagues as our prior work had demonstrated that pharmacists regarded ADE identification, documentation, and reporting to be central to their role, whereas physicians referred patients with ADEs to pharmacists for these tasks. The research assistant shadowed clinical pharmacists in 2 hospital settings in 2- to 4-hour shifts. The research assistant provided the pharmacists a paper version of the ADE reporting form at the beginning of their shifts and asked them to complete it when they encountered an event. The research assistant collected data on the process of completing the form, as well as additional information about the ADE and the relevant workflow.

## Results

We identified 108 active ADE reporting systems worldwide through our systematic review containing 1782 unique data fields [16]. We sorted the data fields into 33 reporting concepts that described the reporter, information about the patient, the ADE, and the suspect and concomitant drugs [16]. We completed 238 hours of observations of clinical pharmacists and 27 hours of observations of physicians in emergency departments, during which care providers encountered 65 possible ADEs [21]. We conducted 12 workshops with over 120 care providers: 6 with hospital pharmacists, 1 with community pharmacists, 2 with emergency department physicians, 1 with general practitioners, and 2 with hospitalists. We completed 25 hours of clinical observations during the pilot-testing phase, which included the documentation of 24 ADEs [18].

Table 1 summarizes the set of data fields that clinicians considered necessary when communicating information about ADEs, along with the formats, value sets, and data sources that they felt were most appropriate or expedient. Some value sets, including those for the data fields “Practitioner Role” and “Level of Certainty,” were drawn from Fast Healthcare Interoperability Resources, which provides standards for data elements created by Health Level Seven International, a health care standards organization. Another source for the value sets was the Medical Dictionary for Regulatory Activities (MedDRA), an international dictionary for a medical terminology that has been clinically validated, applied using the Preferred Terms, which are descriptors for symptoms, diagnosis, and indication. MedDRA allows terms to be mapped to another internationally recognized standard, SNOMED CT, using the Unified Medical Language System metathesaurus.

**Table 1:** Data fields deemed relevant and necessary for adverse drug event reporting by clinicians.

Data Field		Format	Description or Value Set
**Patient Information**			
	Date of birth	Alpha or Numeric	Autopopulate from EMR^a^ or other electronic system, DD-MMM YYYY (eg, 01-FEB 1997)
	Gender	Value set	Autopopulate from EMR or other electronic system (Male or Female or Other or Unknown) as per FHIR^b^
	Name	Alpha	Autopopulate from EMR or other electronic system
	Personal Health Number	Numeric	Autopopulate from EMR or other electronic system
**Reporter Information**			
	Name	Alpha	Autopopulate from EMR login, or entered by clinician
	Practitioner role	Value set	Autopopulate from EMR login, or entered by clinician (Doctor or Nurse or Pharmacist) as per FHIR
	Hospital name and department	Alpha	Autopopulate from EMR, abbreviated
**ADE^c^ Information**			
	Date of report	Alpha or Numeric	Autopopulate from EMR, DD-MMM YYYY (eg, 01-FEB 1997)
	ADE type	Value set	(Adverse drug reaction, Allergy, Incorrect drug, Subtherapeutic dose, Supratherapeutic dose, Treatment failure, Drug withdrawal, Drug interaction, Nonadherence, Other) derived from results of 4 prior prospective studies [8,23-25]
	Symptom caused or exacerbated by ADE	Value set	Predictive entry from MedDRA^d^ Preferred Terms
	Diagnosis caused or exacerbated by ADE	Value set	Predictive entry from MedDRA Preferred Terms
	Relevant tests or lab data (include dates)	Free text	Option for clinician to import from EMR (ideal) or enter manually
	Outcome caused by ADE	Value set	(Death, Permanent disability, Exacerbated pre-existing condition, Congenital anomaly, Hospitalization, Emergency Department visit, Other, Unknown) derived from Health Canada Adverse Drug Reaction reporting standards, amended to reflect qualitative results)—not mutually exclusive
	What happened after dechallenge or treatment?	Value set	(Resolved, Recovering, Ongoing, Resolved with Sequelae, Fatal, Unknown) as per FHIR
	Level of certainty that the adverse event was caused by the suspect drug(s)	Value set	(Certain, Probably or Likely, Possible, Unlikely, Conditional or Classified, Unassessable or Unclassifiable, Refute) as per FHIR
**ADE Treatment Information**			
	Suspect drug actions	Value set	(Discontinue, Change dose, No change)
	Add new medication		Multiple fields (suspect drug or product name, dose, route, frequency, other information)—see Health Product data fields below
	Treatment Status	Value set	(Ordered, Recommended, Received)
**Health Product**			
	Suspect drug or product name(s)	Value set	Option to select from patient's medication list in EMR (ideal) or predictive entry from Canadian Clinical Drug Dataset combined with the Licensed National Health Products Database. Drugs included in the provincial formulary prioritized in search results. Drugs must also be searchable using a DIN^e^ or NPN^f^. Multiple products may be entered as suspect drugs for the same event.
	Dose taken or received	Alpha or Numeric or Special	Manual entry
	Dose unit	Value set	(g, mg, mcg, IU^g^, Units)
	Route of administration	Value set	(Oral, SC^h^, IM^i^, IV^j^, Topical)
	Frequency taken or received	Alpha or Numeric or Special	Manual entry
	Indication for drug	Value set	Prescription indication for use subset developed by Canada Health Infoway [26]
	Other dosing information	Free text	Manual entry
**Other**			
	Additional information (important details or context, timelines, follow-up)	Free text	For clinicians to provide additional information about any of the above, specify follow-up.

^a^EMR: electronic medical record.

^b^FHIR: Fast Healthcare Interoperability Resources.

^c^ADE: adverse drug event.

^d^MedDRA: Medical Dictionary for Regulatory Activities.

^e^DIN: Drug Identification Number.

^f^NPN: Natural Product Number.

^g^IU: International Unit.

^h^SC: subcutaneous.

^i^IM: intramuscular.

^j^IV: intravenous.

Clinicians discussed the tradeoffs of various data formats, noting that structured documentation eliminated confusing shorthand and led to more succinct, comprehensible reports and analyzable data. However, they also noted that they were unwilling to use forms where the data formats forced them to enter inaccurate or incomplete information. Clinicians expressed frustration with value sets that were incomplete or where only one option could be selected when several were relevant (eg, if they had to choose a single symptom or ADE type that did not accurately reflect their patient’s situation or the use of an allergy field for documenting a drug-disease state interaction). Many health outcomes are not mutually exclusive. Therefore, clinicians are able to select more than one health outcome from the list (see “Outcome caused by ADE” field in Table 1). Clinicians noted that many events were not straightforward and required free text to provide important details, context, and follow-up information. Thus, despite the recognition that the use of free text fields can lead to the use of nonstandardized terminology, clinicians felt that a general free text field to enter additional information was needed.

Clinicians highlighted the importance of knowing whether an ADE was treated, and if so, how (see “ADE Treatment Information” fields in Table 1). They were particularly interested in the previous provider’s actions related to the culprit medication: Was it discontinued? Was the dosage changed? Was it replaced? For clinicians, this information was crucial to determining the patient’s medication regimen going forward and avoiding dangerous re-exposure while seeking alternative treatment(s) for the culprit drug’s indication. One design option that was advanced was to link treatment data fields to the physician order page in the electronic medical record to allow physicians to document the event and initiate treatment using the same process.

Clinicians pointed out that a chief concern surrounding ADE documentation was that causality is often uncertain. They needed to be able to indicate their level of certainty regarding the causality of an ADE (see “Level of certainty” field in Table 1). They suggested that it would likely be more accurate for the clinicians to record the certainty or uncertainty of their causality assessment when entering data compared with that completed retrospectively by a data analyst reviewing the report who would lack the immediate knowledge of the patient’s condition and circumstances of the event, as is commonly done in many pharmacosurveillance organizations. Our observations demonstrated that the limited certainty of patients’ medical and medication histories led clinicians to manage patients based on a working, rather than definitive, diagnosis and that ADEs were diagnosed over time and across care settings. Thus, the report, including the level of certainty, is to be a living document with the capacity to edit, update, and refute information by multiple clinicians as information becomes available or as a patient’s condition evolves. We propose that the definitions of this category should be readily available within any electronic system that uses this category to ensure a consistent use.

**Table 2:** Data fields from the existing adverse drug event reporting forms that clinicians felt should be omitted.

Data field		Justification for excluding
**Patient Information**		
	Height or weight	Future providers can obtain this information from patients or their records. Height and weight may be relevant to dosing, but are not essential for assessing most ADEs^a^ and patients’ medication regimen.
	Medical history or concomitant disease states	Burdensome to enter, especially for complex patients. Future providers can often obtain this information from patients or their records.
**Reporter Information**		
	Phone or mailing address or email	Burdensome to enter. If future providers have the reporter’s name, role, and institution, they will likely be able to find the contact information online.
**ADE Information**		
	Reaction start or end date, duration	Can be difficult to pinpoint (eg, delirium). Free text description of timelines is more accurate and in line with clinician charting practices.
	Severity or seriousness	Even with standardized definitions, severity or seriousness assessments are often subjective, differ across contexts, and are prone to error. This information may be better communicated via other fields such as the patient’s outcome (eg, was the patient hospitalized?), their treatment (did the ADE require treatment? Was the drug discontinued?), symptom or diagnosis (eg, anaphylactic reaction or upset stomach), lab data (eg, low sodium of 115 or 125), and dechallenge results (resolved or worsening).
	Rechallenge information	Often unavailable at the point-of-care, or impractical, unethical, or harmful to re-expose the patient intentionally.
**Health Product(s)**		
	Prescribed dose or frequency	The dose prescribed is less relevant than the dose that the patient actually took or received in relation to the ADE. Prescription information can be accessed elsewhere if needed.
	Product strength	The dosage taken by the patient is more important. Given the product name or DIN^b^ or NPN^c^, product strength can usually be obtained.
	Source (eg, pharmacy, grocery store, internet, other)	Generally not essential for assessing the ADE and the patient’s medication regimen.
	Product start or end date, duration	Can be difficult to accurately collect (must rely on patient memory or prescription records that may be unavailable or inaccurate). Free text description of timelines is more accurate and in line with clinician charting practices.
	Manufacturer	Not essential for assessing the ADE and the patient’s medication regimen.
	Batch or lot #	Burdensome to gather; will often require tracing to pharmacy. Very rarely essential for assessing the ADE and the patient’s medication regimen.
	Concomitant health products	Providers should be able to enter multiple suspect drugs, but a complete account of the patient’s medication regimen is burdensome to enter, especially for complex patients. Future providers can usually obtain other medication information from the patient, their records, or by linkage to electronic medication dispensing information depending on the jurisdiction where care is provided.

^a^ADE: adverse drug event.

^b^DIN: Drug Identification Number.

^c^NPN: Natural Product Number.

Table 2 provides an overview of some of the fields that were regularly included in ADE documentation as well as reporting forms encountered by us in our systematic review that clinicians felt could be excluded from our recommended data field set. Many of the fields in Table 2 exist for pharmacosurveillance purposes, including retrospective causality assessments. Clinicians rejected many of these fields, in part because they were skeptical about the accuracy of such retrospective assessments compared with the immediate assessment of the treating clinician. For the purposes of information sharing about ADEs, an indication of the treating clinician’s certainty was seen as more important and required less data entry. Clinicians also rejected data fields such as the manufacturer, batch or lot number, and source, noting that these fields were infrequently available at the point-of-care and clinically irrelevant. Additionally, these fields exist to enable pharmacosurveillance agencies to detect deficiencies in manufacturer quality control that lead to patient harm, which contributed to none of the ADEs we observed. While clinicians noted that some of the fields in Table 2 might be relevant to specific ADEs, they felt that these fields would be less commonly used, would dissuade from reporting because their inclusion would render the form longer, and would have a lower utility for clinical care. They also noted that for cases where the excluded fields were relevant, the reporter could supply this information in free text in a comment field.

Throughout our work, clinicians stressed that duplicate documentation of work was a problem with existing ADE reporting forms, which took time away from patient care activities. They argued in favor of a reporting form that was integrated into the local electronic medical record and could be prepopulated with reporter information (associated with their user account), patient information (associated with the patient’s file), and possibly drug and dosing information (associated with the patient’s medication history).

Discussions with clinicians emphasized striking a balance between too little and too much information. Clinicians felt that ADE documentation should be comprehensive enough to be clinically useful and not require future providers to seek out further information (eg, a documented allergy without enough information can complicate clinical decision making). At the same time, clinicians noted that in complex cases, they might be overwhelmed with the amount of information needed to keep track of a suspect ADE, until such time as a definitive ADE diagnosis could be made. Clinical utility, simplicity, convenience, and, to a lesser degree, signal generation were central considerations for clinicians when refining the set of data fields.

When observing clinical pharmacists pilot-tested the preliminary ADE reporting form containing the data field set developed by us, they felt that its length and level of detail were appropriate. They provided few important suggestions to abbreviate the dataset further. For example, they noted that the “Date of Last Dispense” field was irrelevant to clinical care and could be omitted and that “Follow-up Items” could be noted under “Additional Comments” [18]. Both of these fields were, therefore, removed.

## Discussion

### Principal Findings

Our objective was to describe a set of data fields for clinicians to document and communicate ADEs from the point-of-care to support clinical decision making and improve patient safety. We were able to take a large number of nonstandardized data fields currently in use by ADE reporting systems internationally and condense them to one standardized dataset, while mapping some required fields to internationally recognized standards. In this process, we had to make exclusions and tradeoffs. While not all participating clinicians agreed on every field, our iterative process engaged different types of end users and was far more inclusive than is customary in information technology design in health care.

We recognize that the omission of regulatory fields may be controversial. We have taken this approach from the perspective that clinical tools need to be designed foremost to enhance the immediate delivery of care. Incomplete and nonstandardized information sharing about ADEs across health care sectors and between care providers puts patients at risk [11,12]. However, there are other important reasons why the exclusions of regulatory fields may be justified and beneficial.

First, given the complexity of the ADEs observed by our team, the immediate clinician’s assessment is likely more reliable than a retrospective, at-a-distance assessment. In addition, clinicians preferred to provide causality data from the point-of-care as this assessment was felt to be crucial for informing subsequent clinical decisions.

Second, it was clear that clinicians regarded data fields used to support retrospective information gathering for regulatory agencies as burdensome. To obtain information related to manufacturer quality control issues, such as batch and lot number, clinicians often must attempt to trace the drug back to the pharmacy, a time-consuming activity that is irrelevant for most ADEs. Similarly, fields gathering information already contained in the electronic format prior to the ADE assessment, such as concomitant therapies or product start and end dates, require clinicians to duplicate the entry of information that exists elsewhere. If regulatory assessments require this data, it may be more effective to establish links to complementary datasets (such as prescribing information in a jurisdictional drug information system) than to request that clinicians provide it. We may, simply by easing documentation burden, see an increase in ADE reporting, which would contribute to improved data compared with conventional systems that most clinicians reported never having accessed.

As health systems internationally struggle to motivate providers to report ADEs and new electronic infrastructures are established to improve health information sharing across settings through e-prescribing or drug information systems, our results are timely. New electronic systems offer the potential to streamline information gathering and data entry and consolidate the multiple forms, platforms, information sources, and medication ordering tools that are necessary for clinical work. However, in practice, these systems have the potential to increase documentation burden on clinicians, cause unexpected errors, and desensitize clinicians to alerts due to overflagging and alert fatigue [1,21,27-30]. We see an opportunity to capitalize on new technology by integrating ADE documentation into the systems that clinicians already use, incorporating reporting into clinical workflow, and avoiding duplicate data entry. Clinicians who prescribe and dispense medications expressed interest in using patient-specific ADE data to create patient-specific, medication-level alerts to help them avoid unintentionally re-exposing a patient to the same drug that previously caused harm.

While the selection of standardized data fields alone cannot guarantee the generation of high-quality ADE reports, a clinician-informed design is more likely to result in relevant data. Implementation strategies for this dataset should continue to seek input from clinicians to facilitate uptake and adoption and ensure end-user engagement and adaptation to local contexts. If implemented with attention to clinical workflow, standardized and clinically relevant data fields may yield more accurate and complete information that can inform clinical care and improve patient safety while providing higher-quality representative data for surveillance and research activities.

At the time of publication, our team has programmed this set of data fields into an electronic app, called *ActionADE*, which is being pilot implemented on iPads in a teaching hospital in Vancouver, BC. Plans are underway for its integration with the provincial drug information system so that standardized ADE data can be communicated between providers and across health settings. The piloting and implementation phases of *ActionADE* will follow similar methodological rigor as undertaken in the development phase of the data fields. Throughout our work, our team has maintained contact with key national organizations such as Health Canada, Canada Health Infoway, the Institute for Safe Medication Practices, and Accreditation Canada in an effort to increase the likelihood that the data fields identified here will be adopted nationally. If successfully adopted and implemented, researchers and drug regulators may benefit from the data that would be generated as a by-product of safer care.

### Conclusions

Existing electronic systems allow clinicians to document ADEs, but are nonstandardized and provide limited information that can be shared across health settings and between providers. The structured and standardized set of data fields presented by us are intended to meet the needs of frontline clinicians while enabling a standardized, unambiguous communication between care providers and electronic systems to increase care quality and safety. If implemented, the minimum required data fields have the potential to address the informational discontinuity and reduce ADEs while improving the available health data for pharmacosurveillance and research purposes as a by-product of safer care.

## Abbreviations

ADE: adverse drug event
BC: British Columbia
DIN: Drug Identification Number
EMR: electronic medical record
FHIR: Fast Healthcare Interoperability Resources
IM: intramuscular
IU: International Unit
IV: intravenous
MedDRA: Medical Dictionary for Regulatory Activities
NPN: Natural Product Number
SC: subcutaneous

## Appendix

Multimedia Appendix 1 Excerpt of adverse drug event (ADE) concepts and data fields found during our systematic review using Inspiration 9.2.
