# Supplementary figures and images for: Adverse Drug Event Reporting From Clinical Care: Mixed-Methods Analysis for a Minimum Required Dataset
Source: JMIR Med Inform. 2018 Jun 28;6(2):e10248. doi: 10.2196/10248 (PMC6043729; doi:10.2196/10248)

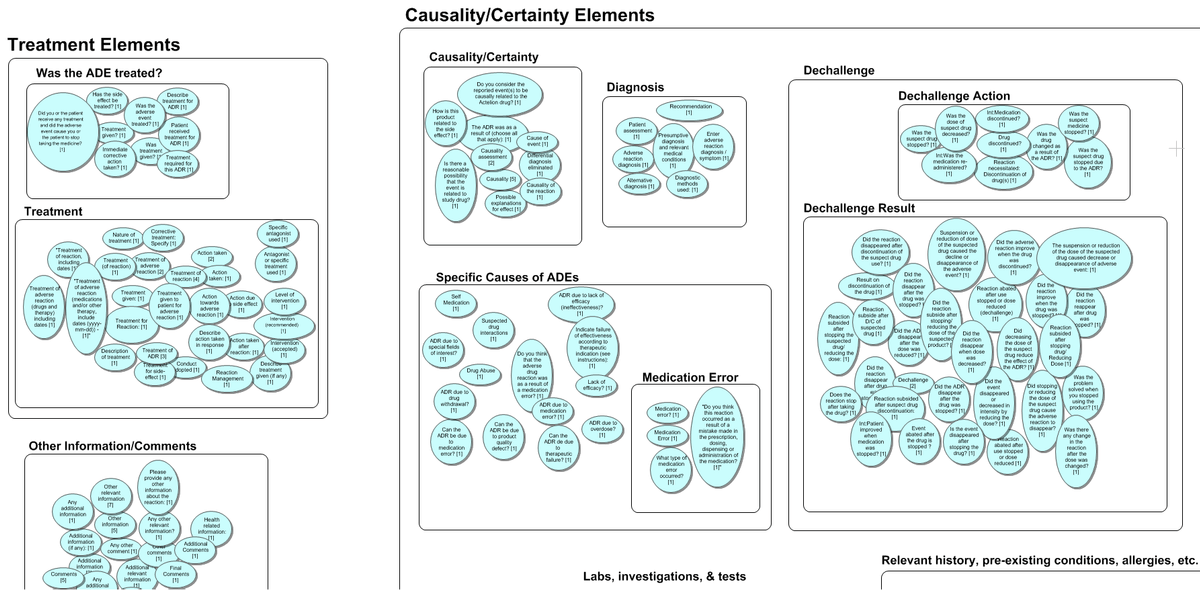

Supplement: Multimedia Appendix 1 [file medinform_v6i2e10248_app1.png]
